# Co-delivery of docetaxel and palmitoyl ascorbate by liposome for enhanced synergistic antitumor efficacy

**DOI:** 10.1038/srep38787

**Published:** 2016-12-09

**Authors:** Junxiu Li, Chaorui Guo, Fan Feng, Ali Fan, Yu Dai, Ning Li, Di Zhao, Xijing Chen, Yang Lu

**Affiliations:** 1 Clinical Pharmacokinetics Laboratory, School of Basic Medicine and Clinical Pharmacy, China Pharmaceutical University, #639 Longmain Avenue, Jiangning District, Najing, 211198, China

## Abstract

Palmitoyl ascorbate (PA) as an antioxidant has the potential for the treatment of cancer. In the present study, a nanocarrier system was developed for co-delivery of docetaxel (DOC) with palmitoyl ascorbate and the therapeutic efficacy of a combination drug regimen was investigated. For this purpose, different ratios of docetaxel and palmitoyl ascorbate were co-encapsulated in a liposome and they all showed high encapsulation efficiency. The average diameters of the liposomes ranged from 140 to 170 nm. Negative zeta potential values were observed for all systems, ranged from −40 mV to −56 mV. Studies on drug release and cellular uptake of the co-delivery system demonstrated that both drugs were effectively taken up by the cells and released slowly. Moreover, the liposome loading drugs with DOC/PA concentration ratio of 1:200 showed the highest anti-tumor activity to three different types of tumor cells. The higher *in vivo* therapeutic efficacy with lower systemic toxicity of the DOC-PA_200_-LPs was also verified by the H22 tumor bearing mice model. Our results showed that such co-loaded delivery systems could serve as a promising therapeutic approach to improve clinical outcomes against hepatic carcinoma.

Ascorbate plays an important role in various functions of living organisms. Recent years, a large number of evidences have increasingly supported the hypothesis that high dose ascorbate potently kills cancer cells *in vitro* and in cancer-bearing animals *in vivo*^1^,^2^,^3^. The mechanism of its anticancer effect is mainly attributed to the production of tumoricidal hydrogen peroxide^4^. It may also include stimulatory effects on apoptotic pathways, by accelerating pro-oxidant damage that cannot be repaired by tumor cells and increasing oxidation of ascorbate at high concentration which in turn can be toxic^5^.

Ascorbate has been well documented to reduce the incidence of most malignancies in humans, but most of the treatments require high-dose ascorbate^6^,^7^,^8^,^9^. Recent pharmacological experiment reports have revealed that orally administered ascorbate, even at very large and frequent dose, marginally increases plasma concentrations from 0.07 mM to a maximum of 0.22 mM^10^. Conversely, intravenous ascorbate administration can raise plasma concentrations as high as 14 mM, and concentrations of 1–5 mM have been found to be selectively cytotoxic to tumor cells *in vitro*^11^. The pharmacokinetic study of ascorbate has provided valuable information regarding plasma ascorbate concentrations with different routes of administration^12^. Now it is clear that intravenous administration of ascorbate could result in high plasma levels, while oral treatment does not.

As a single agent, only extremely high dose of ascorbate shows anticancer effect. Thus, it appears promising to co-deliver ascorbate with a chemotherapy agent. To increase the activity, the use of i.v. ascorbate in combination with cytotoxic chemotherapy is further encouraged. Ascorbate has shown to increase the therapeutic effect of various *in vitro* anticancer treatments with improved efficacy of gemcitabine, cisplatin, and paclitaxel in cancer cell^13^,^14^,^15^ and has been studied for the use with motexafin gadolinium^16^ and arsenic trioxide^17^. Also, it could increase the cytotoxicity of vincristine on non-small-cell lung cancer cells, reversing their vincristine resistance^18^.

The instability of aqueous ascorbate also has promoted a number of investigators to seek a more stable ascorbate derivative^19^. Ascorbate and its derivatives were shown to be cytotoxic and inhibited the growth of a number of malignant and non-malignant cell lines *in vitro* and *in vivo*^20^. Among these ascorbate derivatives, palmitoyl ascorbate (PA) is often used in topical preparations against oxidative changes of biological components of the skin, and as an antioxidant to protect lipophilic ingredients in formulations^21^. Compared with ascorbate, PA also shows stronger inhibition on the growth of cancer cells and prolong the lifespan of tumor bearing mice^22^. It may alter DNA synthesis and the proliferation of tumor cells, leading to the release of membrane phospholipids and suppressing the post-transcriptional induction of ornithine decarboxylase^23^.

Liposomes with the diameter in nano scale have been widely used for drug delivery to enhance solubility, permeability and stability of drugs while minimizing systemic toxicity. PA is almost insoluble in water at room temperature, and liposomes are a good choice for PA to deliver into tumor^24^,^25^. Moreover, the chemical stability of PA was reported to be enhanced when it was encapsulated in solid lipid nanoparticles^26^,^27^. The aim of present study was to co-deliver PA and docetaxel (DOC) via a liposome-encapsulated nanoparticles system and evaluate its anticancer effect in MCF-7, HepG-2, PC-3 cell lines. The tumor studies were also carried out using the tumor bearing mice with H22 hepatoma cells. The results indicate that co-delivery PA with docetaxel liposome may be considered as an attractive and promising formulation for liver cancer treatment.

## Materials and Methods

### Materials, cell lines, and animals

Docetaxel (purity 99%) and palmitoyl ascorbate (purity 99%) were obtained from Shanghai Sunve Pharmaceutical Co. Ltd, Wuhan Dahua Pharmaceutical Co. Ltd, respectively. Phosphatidylcholine from soybean (purity 90%) and cholesterol (purity 95%) were purchased from Shanghai Taiwei Pharmaceutical Co. Ltd and Shanghai J&K Scientific Co. Ltd, respectively. All other chemicals were of analytical grade. 3-(4,5-dimethyl-2-thiazolyl)-2,5-diphenyl tetrazolium bromide (MTT) reagent, 4,6-diamidino-2-phenylindole (DAPI), and coumarin-6 were purchased from Sigma-Aldrich (Shanghai, China). Cell culture were cultured in Dulbecco’s Modified Eagle Medium (DMEM), trypsin-EDTA, penicillin, streptomycin, and fetal bovine serum (FBS) and they were provided from HyClone Labs Inc. Murine H22 hepatoma cells were obtained from China Institute of Cell Biology, maintained in DMEM medium supplemented with 10% fetal bovine serum.

ICR (Institute of Cancer Research) mice (18–20 g) were purchased from the Shanghai SIPPR/BK Experiment Animal Company Ltd.(China). All the animals were free to access food and water during the whole experiment. To generate the tumor model, the mice were inoculated with 2 × 10^6^ viable cells subcutaneously on the dorsum. All protocols were evaluated and approved by the Ethics Committee for Animal Experimentation of China Pharmaceutical University (Nanjing, China). All efforts were made to minimize animal’s discomfort and to reduce the number of animals used.

### Liposome Preparation

The liposomes were prepared using a thin-film hydration technique. Briefly, 60 mg PA, 225 mg phosphatidylcholine and 75 mg cholesterol were dissolved in 15 ml dichloromethane. The solvent was evaporated to dryness using a rotary evaporator under reduced pressure at 30 °C, producing a thin lipid film. The film was placed in a cabin drier overnight to remove the residual solvent. Then the lipid film was rehydrated with the purified water, and followed by ultrasonication with three cycles at 4 °C. The PA at different weight ratio to docetaxel (20:1, 50:1, 100:1, 200:1) were prepared in the same method, and they were named as DOC-PA_20_-LPs, DOC-PA_50_-LPs, DOC-PA_100_-LPs, DOC-PA_200_-LPs respectively.

### Measurement of particle size, ζ-potential, and morphology

The particle size, polydispersity index (PDI), and ζ-potential were analyzed using a Dynamic Light Scattering Analyzer (Brookhaven, USA) and a Zeta Plus Zeta Potential Analyzer (Brookhaven, USA) respectively. The morphology of the liposome formulation was examined using a transmission electron microscope (TEM, Tokyo, Japan). A drop of dispersed liposome was stratified into a carbon coated grid and left to adhere onto the carbon substrate for about 30 min. The excess was removed, and phosphotungistic acid hydrate was added. After drying, the grid was observed using TEM at an accelerating voltage of 100 kV.

### Measurement of drug loading and encapsulation efficiency

The entrapment efficiency was calculated by the following equation: EE = W_o_/W_i_ × 100%, where W_o_ and W_i_ are the amount of drug before and after ultrafiltration.

To evaluate the non-encapsulated PA and DOC, 500 μl of the liposomal formulation was filtered via centrifugal ultrafiltration (MWCO 3 KD, Millipore, USA) for 30 min at 15000 rpm. The content of DOC and PA in liposome was assayed by LC-MS/MS. Mass spectrometric analysis was performed on a triple quadrupole mass spectrometer operated in the multiple reaction monitoring (MRM) mode with the transitions of m/z 830.0→549.0 for quantification of DOC and m/z 413.2→254.8 for quantification of PA. A reverse phase HPLC column (Hypersil GOLD C18:100 × 2.1 mm) was used. The flow rate was set as 0.3 ml/min and the column temperature was set as 25 °C.

### *In vitro* drug release assay

The dialysis bag diffusion technique was employed to investigate the *in vitro* drug release from the liposomes. Briefly, 4 ml of liposome was placed in the dialysis bag (MWCO 14 kDa) and immersed into 30 ml of the NaH_2_PO_4_/Na_2_HPO_4_ buffer solution (10 mM, pH6.8) containing 1% (v:v) Tween 80. The entire system was kept at 37 °C with continuous shaking at 75 rpm. At predetermined time interval, 1 ml of samples were withdrawn and replaced with fresh buffer solution. The analysis procedure was similar with the measurement of EE.

### Evaluation of synergism between PA and docetaxel at various ratios

The synergistic anticancer efficacy of PA in combination with docetaxel *in vitro* was tested in MCF-7, HepG2 and PC-3 cancer cells. Cells (5 × 10^3^) were seeded in 96-well plates, incubated overnight at 37 °C, and treated with the DOC or PA individually and the four formulations (DOC-PA_20_-LPs, DOC-PA_50_-LPs, DOC-PA_100_-LPs, DOC-PA_200_-LPs). After 24 h, cell viability was assessed by MTT assay. The cells were washed with PBS twice and maintained in DMEM medium with 10% fetal bovine serum (FBS) and 1% penicillin/streptomysin. MTT regent (5 mg/ml) was added to each well, and the samples were incubated for 4 h at 37 °C in the dark. The cells were then lysed, and the formazan crystals were dissolved in 150 μl of DMSO. The absorbance was measured at 490 nm using a microplate reader, and the combination ratio was evaluated.

### Analysis of synergism or antagonism *in vitro*

Median effect analysis using the combination index (CI) method of Chou and Talalay^28^ was used to determine the interaction between DOC and PA. The CI value is defined by the following equation: CI = D_A_/D_x,A_ + D_B_/D_x,__B_, where D_A_ and D_B_ are the concentrations of drug A and drug B used in combination to achieve x% drug effect. D_x,A_ and D_x,B_ are the concentrations for single agents to achieve the same effect. CI > 1 indicates antagonism, CI < 1 indicates synergy, and CI = 1 indicates additivity. Each CI ratio represented here is the mean value derived from at least three independent experiments. The *in vitro* drug-induced cytotoxic effects were measured by the MTT reduction assay as mentioned above.

### Confocal microscopy

To investigate *in vitro* cellular uptake, the cellular uptake of PA liposome, DOC was replaced with a hydeophobic probe coumarin-6 exhibiting green autofluorescence, which was entrapped in the same preparation procedure. MCF-7 cells were seeded in 24 well plate at a density of 1 × 10^5^ cells/well and incubated for 12 h in a CO_2_ incubator (5% CO_2_ at 37 °C). Afterwards, cells were incubated with C6-loaded PA liposome at concentration of 4 mg/ml and incubated for 4 h. At desired time points, the nuclei were stained with DAPI (blue). The cells were rinsed with PBS and exposed to confocal microscopy examination.

### *In-vivo* antitumor activity

The study protocol was approved by the Animal Use Committee of China Pharmaceutical University (Nanjing, China) and was carried out in accordance to with the guideline of experimental animals of China Pharmaceutical University. To evaluate the antitumor activity of liposomal DOC, PA and DOC-PA_200_-LPs *in vivo*, tumor-bearing mice were randomly divided into three groups (8 mice each group). Mice in each group were intratumorally administrated normal saline, PA liposome, DOC liposome and DOC-PA_200_-LPs, at DOC dose of 0.5 mg/kg and PA dose of 20 mg/kg. Tumor sizes and body weights were recorded every 3 days. The tumor diameters were measured on alternate days with a vernier caliper in two dimensions. Individual tumor volumes (V) were calculated using the formula: V = (L × W^2^)/2, where in length (L) is the longest diameter and width (W) is the shortest diameter perpendicular to length. After the treatment, the mice were sacrificed and the tumors were harvested for histological analysis using the hematoxylin and eosin (HE) staining.

### Statistical analysis

Data were presented as mean ± SD. Statistical analysis was performed using one-way analysis of variance for multiple samples and Student’s t-test for comparing paired sample sets. A *p* value less than 0.05 was considered statistically significant and *p* < 0.001 was highly significant.

## Results and Discussions

### Characterization of DOC-PA liposomes

**Figure 1:**
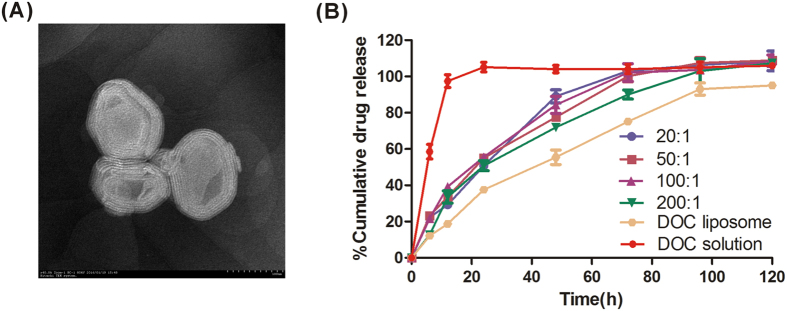
(**A**) TEM image of DOC-PA_200_-LPs. (**B**) The *in vitro* release profile for DOC from liposomes. Each value represents the mean ± SD. (n = 3).

The resultant liposomes were characterized in terms of size and PDI, ζ-potential and surface morphology. The mean diameter and size distribution of liposome appeared to be little different from the results obtained by dynamic light scattering measurement. The mean particle size ranged from 140 to 170 nm for drug individually to dual-drug-loaded liposome and relatively uniform. Zeta potential is another important parameter, indicating the stability of nanocarrier systems. A relatively high surface charge may provide a repelling force between the particles, thus they could be more stable in solution due to less aggregation. Table 1 shows that the all the liposomes had high negative zeta potentials of −40 mV due to negatively charged phospholipids^29^. It is reasonable to conclude that the charged particles may repel each other and prevent aggregation or precipitation. TEM observation demonstrated that the shape of co-delivery liposome was near spherical and they were multi-lamellar, as shown in Fig. 1A.

The encapsulation efficiency (EE) of DOC in the liposome was all above 90%. This high entrapment efficiency probably resulted from the good solubilization of the oil phase and surfactants for the drug. DOC is a poorly water-soluble drug, which makes it suitable for dissolution in the oil phase, thereby producing high encapsulation efficiency. PA is a hydrophobic derivative of AA, but it obtains the property of surface activity. The EE for PA was also very high in co-delivery liposomes which confirms DOC and PA can be co-delivered in a single nano-vehicle efficiently.

### *In vitro* release and cellular uptake analysis

It is difficult to maintain a good sink condition for poorly water soluble drugs in designing *in vitro* drug release experiments. In this research, the sink condition was maintained by the addition of Tween-80 and the frequent replacement of same volume of fresh buffer during the experiment. The *in vitro* release profiles of the Docetaxel-loaded liposomes in the first 5 days were shown in Fig. 1B. The DOC solution group showed a burst release, over 40% of DOC release was observed at 6 h and more than 70% of the drug was released after 12 h. DOC and DOC-PA-LPs showed a sustained-release behavior, and the release of DOC liposome was slower than DOC-PA liposomes. The results may indicate PA can accelerate the release speed of DOC. However, there was no significant difference in DOC release among the four ratio co-delivery formulations even at 120 h.

### Cell uptake

**Figure 2:**
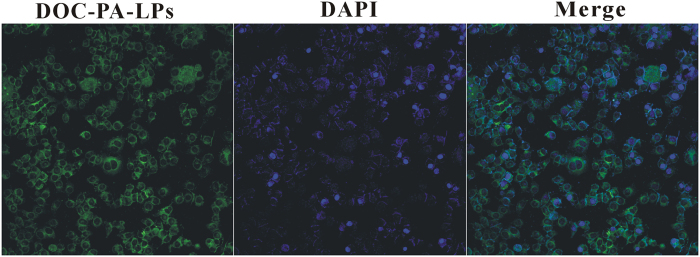
Confocal laser scanning microscopy of MCF-7 cells after 4 h incubation with coumarin 6-loaded PA liposomes at 37 °C.

It was reported that the therapeutic effects of drug-loaded nanoparticles would greatly depend on internalization and sustained retention of nanoparticles by the diseased cells^30^. Coumarin 6, a fluorescence marker, has been extensively studied as a tool for investigating cellular processes due to its advantages such as biocompatibility, high fluorescence activity, low dye loading and low leaking rate^31^. To evaluate cellular uptake of nanoparticles, MCF-7 was incubated with drug-loaded liposomes. The fluorescence emitted by coumarin-6 was observed after liposomes incubation (Fig. 2), which suggested that the liposomes entered the cells successfully.

### *In vitro* synergistic cytotoxicity of DOC and PA at various ratios

**Figure 3:**
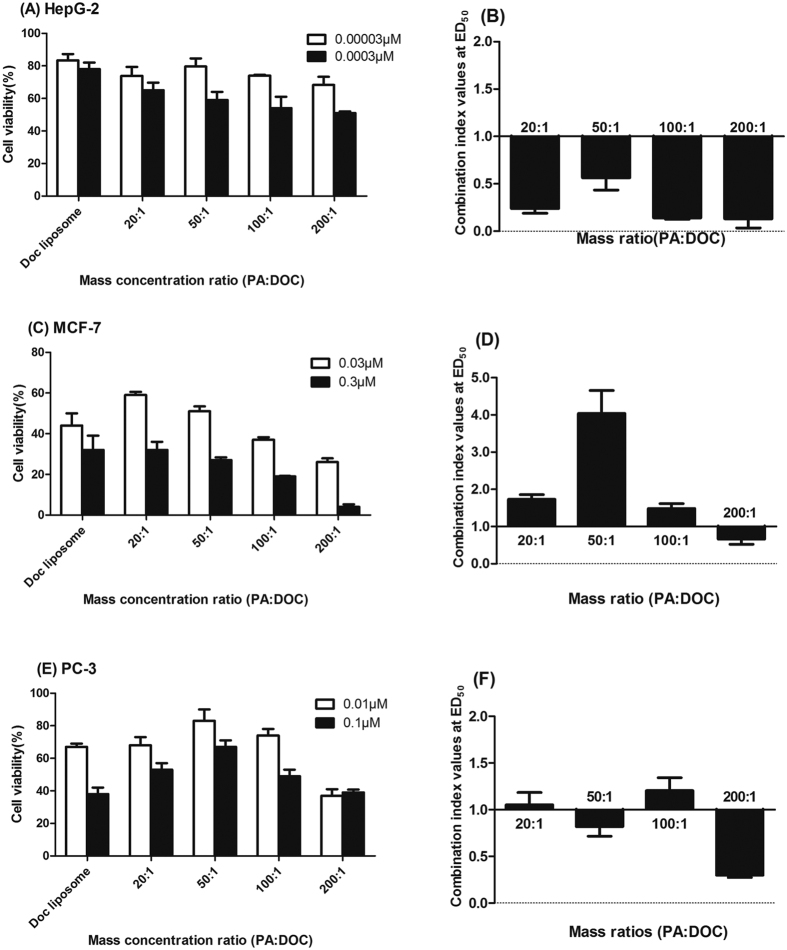
*In vitro* cytotoxicity assays of DOC and DOC-PA-LPs at the indicated ratios in (**A**) HepG2, (**C**) MCF-7, (**E**) PC-3 cells. The values at the median effective dose for DOC and PA combined at the weight ratios of 20:1, 50:1, 100:1, 200:1 are shown for (**B**) HepG2, (**D**) MCF-7, (**F**) PC-3 cells.

The cytotoxicity experiment was performed in MCF-7, HepG2, PC-3 cancer cells via MTT assay. It can be observed that DOC-PA-LPs shows better therapeutic efficacy in HepG2 cells than in other cell lines. All the four dual drug formulations with different drug ratios showed enhanced anticancer effect than single drug-loaded liposomes in the HepG2 cells (Fig. 3A). Meanwhile, their CI value for all the formulations in HepG2 also showed good synergistic effect (Fig. 3B). However, the co-delivering liposome didn’t show good cytotoxicity in PC-3 and MCF-7 cells and appeared to be antagonistic effect (Fig. 3C,E). Except that the CI value of DOC-PA_50_-LPs in PC-3 cancer cells was slightly less than 1, all other formulation produced antagonistic effect in both MCF-7 and PC-3 cells in varying degree (Fig. 3D,F). Nevertheless, along with the increased percentage of PA, the cell viability progressively decreased and the synergism gradually appeared or enhanced. With this tendency, 200:1 finally significantly reduced all cells proliferation and showed a very great synergistic effect in all cell lines (0.28 for PC-3, 0.66 for MCF-7 and 0.13 for HepG2), achieving a noticeably synergistic inhibitory effect through simultaneous delivery of PA with DOC at a low concentration.

### *In vivo* studies

**Figure 4:**
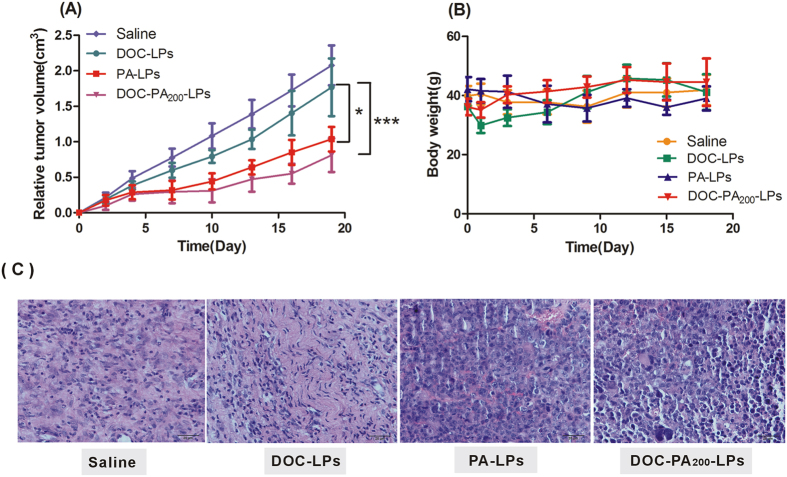
Relative tumor volume (**A**) and body weights (**B**) of H22-bearing mice treated with saline, DOC liposome, PA liposome, DOC-PA_200_-LPs after a schedule of multiple doses. The differences among different groups were significant (***p < 0.001,n = 8). (**C**) Hematoxylin and eosin staining of tumor sections from mice treated with saline, DOC liposome, PA liposome and DOC-PA_200_-LPs.

Tumor-bearing mice models were used for the evaluation of the *in vivo* anti-tumor efficacy of DOC, PA and DOC-PA liposomes. As shown in Fig. 4A, tumor volume of saline treatment group increased rapidly, while the chemotherapy groups were effective in tumor regression. Notably, the maximum antitumor effect was observed in mice treated with DOC-PA liposome, indicating that this combination therapy produced significant effective inhibition of tumor growth (p < 0.001). An analysis of body weight variations generally defined the safety of the different therapy regiments (Fig. 4B). These findings indicated the DOC-PA liposome showed a significant antitumor activity in H22 tumor mice, with a very low systemic toxicity. As shown in Fig. 4C, the histopathological analysis further confirmed the effective tumor inhibition caused by the combination of co-encapsulated DOC and PA as DOC-PA_200_-LPs induced cell karyolysis and apoptosis.

## Discussion

In the last decade, several studies have reported the cancer-specific toxicity of pharmacological ascorbate delayed tumor growth in laboratory studies and delayed tumor growth in rodent xenograft models. While as a single agent, high-dose ascorbate does not demonstrate anticancer activity in clinical trials^32^. However, ascorbate could be a useful adjuvant to existing therapy as a combination agent because of its low toxicity profile. Recently, a few derivatives of ascorbate were tested on cancer cell, among them palmitoyl ascorbate showed promising anticancer activity compared to ascorbate^22^. Also the 6-O-palmitoyl derivative of ascorbate has been demonstrated to exert cytotoxicity to tumor cells through hydrogen peroxide generation. And the latest research efforts in this field now focus on combining this palmitoyl derivative of ascorbate with other cytotoxic drugs and ionizing radiation^25^,^33^. Though that potential synergetic anti-cancer effect has been studied, none of those studies have reported about co-delivering docetaxel and PA.

Our *in vitro* studies confirmed that PA-loaded liposome were effective for killing tumor cells compared with DOC-LPs. especially in high dose. The cytotoxicity induced by ascorbate seems to be primarily mediated by hydrogen peroxide and superoxide. And the mechanism of action of PA is similar to that of free ascorbate. Further, fluorescent microscopy using ROS-sensitive markers confirmed the generation of ROS after treatment of cells with PA-liposomes. Rupa R. *et al*.^25^ studied the importance of extracellular ROS in the anti-cancer-toxicity by PA-liposomes. They discovered that cell killing by PA-loaded liposomes was prevented by catalase, SOD. Gerard G’s data^33^ also showed that TNF-α enhanced the association of PA liposomes with cancer cells and that the presence of SOD which removes ROS reduced this increase in cell association mediated by TNF-α. These results indicate that anti-cancer activity of PA-loaded liposomes might be inhibited by the absence of the superoxide or if the peroxide is detoxified by catalae.

In our study, it was reasonable to realize that PA can inherit some characteristics from ascorbate due to the fact that PA, as an aclylated derivative of it, was used to deliver ascorbate moieties to cancer cells. It has been shown that the amount of hydrogen peroxide generated by the cells was proportionally dependent on the ascorbate concentration and inhibited by serum. Ascorbate in low concentrations even has been found to be an essential requirement for the growth of cancer cells in cell culture^34^. These studies may suggest PA would have the similar situation in terms of understanding dose effect on cancer cells. Also, the antagonistic effect may always be discovered at a low dose of PA due to this reason. PA was also found to show a dose-dependent toxicity towards a variety of tumor cells as previously reported^33^. Meanwhile, the optimal synergistic effect was always observed at a high toxicity dose of PA^10^. This synergistic effect might have resulted from the different antitumor mechanisms of these compounds. As mentioned above, the cytotoxicity of PA was mediated by H_2_O_2_. Also, DOC obtains the ability of disrupting normal dynamic reorganization of the microtubule network which is required for mitosis and cell proliferation, and in turn causes cell apoptosis^35^. According to the *in vitro* studies, the application of DOC-PA _200_-LPs in the treatment of cancer cells might accelerate tumor cell death and achieve synergistic antitumor effect efficiently.

Gerard G and Rupa R.’ reporters were the only two studies on PA-modified liposmes with paclitaxel, but there was little information about cytotoxicity of co-delivering PA and paclitaxel. In our study, the anticancer effect of DOC-PA-LPs to different types of human tumor cells has been first studied. The result shows that DOC-PA-LPs could act toxicity to HepG2 at a very low concentration. Meanwhile, DOC-PA-LPs performed a synergetic value of CI to HepG2. It may suggest that HepG2 was more sensitive to the toxicity of DOC-PA-LPs than MCF-7 and PC-3. The *in vitro* results of cancer cells treated with DOC-PA_200_-LPs were further confirmed in a H22 tumor mice. The tumor growth followed the order: DOC-PA_200_-LPs < PA-LPs < DOC-LPs < Saline. The DOC-PA_200_-LPs markedly inhibited tumor growth (p < 0.001) and PA-LPs are quite toxic on their own. The results presented here indicated that PA at a much higher dose could be substantial and greater than that of DOC liposomes without PA. A co-treatment effect was clearly observed at a high dose of PA (20 mg/kg). However, the effect of DOC-PA_200_-LPs does not have significant difference with PA-LPs. Maybe at such a high ratio and at such a larger dose, the effect of high PA dose not leave much room for the effect of low DOC dose^25^.

In clinical applications, liposomal drugs have been proven to be the most useful for their ability to “passively” accumulate at sites of increased vasculature permeability and for their ability to reduce the side effects of the encapsulated drugs relative to free drugs^36^. The PA-loaded liposomes described in this study clearly retained the toxicity of ascorbate towards tumor cells. The liposome size of about 140 nm is ideally suited for *in vivo* applications and should allow for selective accumulation in solid tumors by virtue of the well-documented enhanced permeability and retention effect. In this study, a realistic delivery system close to clinical application to satisfy the need was used. The materials innovation and processing of drug delivery systems, which still need a comprehensive evaluation of safety before being put into clinical practice^36^, were not the focus.

## Conclusion

In this study, co-encapsulated DOC-PA-LPs was prepared by a thin-film hydration technique. The prepared DOC-PA-LPs were nanosized (140–170 nm) and showed negative zeta potential, with sustained drug release characteristics. The synergistic antitumor effect of co-delivery liposomes was also evaluated both *in vitro* and *in vivo*. The CI values from *in vitro* cytotoxicity studies demonstrated that the combined use of PA and DOC at a weight ratio of 200:1 had the highest synergistic effect in HepG2, MCF-7 and PC-3 lines. Furthermore, the dual drug PA-DOC liposome suppressed tumor cell growth *in vivo* more efficiently than either PA or DOC individually. Thus, the co-delivery of PA and DOC in one nanocarrier system may provide a promising combined therapeutic strategy for tumor targeting and enhanced antitumor therapy.

## Additional Information

**How to cite this article**: Li, J. *et al*. Co-delivery of docetaxel and palmitoyl ascorbate by liposome for enhanced synergistic antitumor efficacy. *Sci. Rep.* **6**, 38787; doi: 10.1038/srep38787 (2016).

**Publisher's note:** Springer Nature remains neutral with regard to jurisdictional claims in published maps and institutional affiliations.

## Figures and Tables

**Table 1:** Characterization of co-encapsulated DOC and PA liposomes.

	Particle size (nm)	PDI	Zeta potential (mV)	EE% (DOC)	EE (PA)
PA	169.1 ± 1.6	0.248 ± 0.004	−47.3 ± 0.4	\	99.8 ± 0.1
DOC	156.2 ± 1.6	0.233 ± 0.01	−55.6 ± 1.0	99.9 ± 0.02	\
20:1	159.5 ± 1.5	0.207 ± 0.005	−40.7 ± 1.0	97.4 ± 0.3	99.7 ± 0.1
50:1	145.8 ± 1.6	0.257 ± 0.012	−46.9 ± 2.0	98.1 ± 0.03	97.7 ± 0.3
100:1	145.3 ± 1.6	0.215 ± 0.02	−46.7 ± 0.8	97.1 ± 0.2	92.2 ± 0.7
200:1	147.2 ± 1.6	0.212 ± 0.005	−47.2 ± 0.4	96.0 ± 0.9	96.7 ± 0.2
